# Early stage breast cancer treatment and outcome of older patients treated in an oncogeriatric care and a standard care setting: an international comparison

**DOI:** 10.1007/s10549-020-05860-7

**Published:** 2020-08-19

**Authors:** Anna Z. de Boer, Willemien van de Water, Esther Bastiaannet, Nienke A. de Glas, Mandy Kiderlen, Johanneke E. A. Portielje, Martine Extermann

**Affiliations:** 1 Department of Surgery, Leiden University Medical Center, Location J10-71, Postzone K6-R, P.O. Box 9600, 2300 RC Leiden, The Netherlands; 2 Department of Medical Oncology, Leiden University Medical Center, Leiden, The Netherlands; 3 Department of Radiotherapy, Erasmus Medical Center, Rotterdam, The Netherlands; 4 Department of Senior Adult Oncology, H. Lee Moffitt Cancer Center and Research Institute, Tampa, FL USA

**Keywords:** Breast cancer, Geriatric oncology, Oncogeriatric care, Geriatric assessment, Recurrence risk

## Abstract

**Introduction:**

Since older patients with breast cancer are underrepresented in clinical trials, an oncogeriatric approach is advocated to guide treatment decisions. However, the effect on outcomes is unclear. The aim of this study was to compare treatments and outcomes between patients treated in an oncogeriatric and a standard care setting.

**Methods:**

Patients aged ≥ 70 years with early stage breast cancer were included. The *oncogeriatric cohort* comprised unselected patients from the Moffitt Cancer Center, and the *standard cohort* patients from a Dutch population-based cohort. Cox models were used to characterize the influence of care setting on recurrence risk and overall mortality.

**Results:**

Overall, 268 patients were included in the oncogeriatric and 1932 patients in the standard cohort. Patients in the oncogeriatric cohort were slightly younger, had more comorbidity, and received more adjuvant endocrine therapy and chemotherapy. Oncogeriatric care was associated with a lower risk of recurrence, which remained significant after adjustment for patient and tumour characteristics [hazard ratio (HR) 0.66, 95% confidence interval (CI) 0.44–0.99]. Oncogeriatric care was also associated with a lower overall mortality, which also remained significant after adjustment for patient and tumour characteristics (HR 0.69, 95% CI 0.55–0.87).

**Conclusions:**

Patients treated in the oncogeriatric care setting had a lower risk of recurrence, which may be explained by more systemic treatment. Overall mortality was also lower, but other explanations besides care setting could not be ruled out as the cohorts had different patient profiles. Future studies need to clarify the impact of an oncogeriatric approach on outcomes.

**Electronic supplementary material:**

The online version of this article (10.1007/s10549-020-05860-7) contains supplementary material, which is available to authorized users.

## Introduction

The number of older patients with breast cancer is increasing due to ageing of Western societies [1]. Despite the fact that patients aged 70 years or older make up over 30% of all patients with breast cancer, this patient population is underrepresented in clinical trials [2, 3]. As a result, treatment recommendations based on studies with younger patients are currently extrapolated to older patients [4]. However, due to comorbidity and geriatric deficits such as reduced physical and cognitive functioning, not all older patients will experience the same treatment benefits as younger patients. Furthermore, geriatric deficits are predictive for treatment toxicity [5]. Therefore, in order to guide treatment decisions in the heterogeneous population of older patient with breast cancer, the International Society of Geriatric Oncology (SIOG) and European Society of Breast Cancer Specialists (EUSOMA) advocate an oncogeriatric approach with a multi-domain geriatric assessment (GA) [4, 6]. The aim of this approach is to tailor treatment to the individual older patient taking comorbidity and functioning on all geriatric domains into account [4, 6–9].

Studies have demonstrated that a GA can reveal health problems beyond those identified in a standard history taking and physical examination [10, 11]. Second, geriatric parameters have been established that can predict chemotoxicity [12, 13] and estimate residual life expectancy [14]. Last, it has been shown that a GA can alter treatment decisions in general oncology patients [6, 8, 10, 15] as well as in patients with breast cancer specifically [16], although for the latter, previous studies were small. However, evidence on the effect of an oncogeriatric approach on outcomes is lacking [15]. Therefore, the aim of the present study was to compare treatments, risk of recurrence and overall mortality between older patients with early stage breast cancer treated in an oncogeriatric and a standard care setting.

## Methods

### Cohorts

In this retrospective comparative observational study, patients who were diagnosed at the age of 70 years or older with early stage breast cancer (T1-2N0-1) between 1997 and 2004 were included. The *oncogeriatric cohort* comprised a hospital-based cohort of consecutively treated patients at the H. Lee Moffitt Cancer Center and Research Institute in Tampa, Florida, USA. This cohort included patient that were referred after being diagnosed (before undergoing any treatment) in a different hospital. The *standard cohort* comprised a regional population-based cohort of patients treated in the South-West part of the Netherlands identified from the Netherlands Cancer Registry (NCR).

### Description of care

The majority of patients in the oncogeriatric cohort was seen in Moffitt’s Senior Adult Oncology Program (SAOP) [17]. At the first visit, patients underwent a geriatric screening with the SAOP screening tool. In addition to functional, depression and cognitive screening, this tool includes questions on quality of life, self-rated health, falls, nutrition, sleep, polypharmacy and social issues such as drug payment and caregiver availability. If one item was impaired, the concerning specialist was consulted, and if two or more items were impaired, a comprehensive geriatric assessment was initiated. Referrals to concerning specialists, which are amongst others dieticians, pharmacists and social workers, are considered general geriatric interventions. Furthermore, all patients were discussed in a multidisciplinary meeting attended by geriatric oncologists and geriatric nurse practitioners who granted special focus on geriatric parameters, and prediction tools were used as appropriate, for example the CRASH (Chemotherapy Risk Age Scale for High Risk Patients) score which predicts the risk of chemotherapy toxicity [18]. In the standard cohort, no geriatric screening tools or geriatric assessments were performed. All patients were discussed in a multidisciplinary meeting which were not attended by a geriatrician or geriatric nurse. Nor was a geriatrician consulted separately. Follow-up schemes were in line with national guidelines at the time; patients in the oncogeriatric cohort underwent a history taking and physical examination every 6 months for 5 years and every year thereafter, whilst patients in the standard cohort every 3 months in the first 2 years and every year thereafter. In both cohorts a mammography was performed every year.

### Data

Data were retrospectively collected by means of chart review. Tumour information was extracted from pathology reports and based on the tumour-node-metastasis (TNM) classification as used at the time [19, 20]. Clinical stage was used if pathological stage was unknown. Comorbidity at time of diagnosis was categorized by number of categories included in the 10th edition of the International Statistical Classification of Diseases and Related Health Problems [21]. All patients were included at time of diagnosis. In the standard cohort, vital status was available until January 1st 2011 through linkage with municipal population registries. Vital status for the oncogeriatric cohort was established directly from the medical record or through linkage with the National Death Index and was censored at January 1st 2011. Patients who moved out of the region were censored at time of last follow-up visit. Follow-up for recurrence was defined as time from diagnosis until (locoregional or distant) recurrence, last follow-up visit or censoring date, whichever came first. Carcinoma in situ and contralateral breast cancer were not considered a recurrence. Follow-up for vital status was defined as time from diagnosis until death, last follow-up visit or censoring date, whichever came first.

### Statistical analysis

Stata SE 12.0 was used for the statistical analysis. Differences in baseline characteristics between the two cohorts were assessed by means of Pearson’s Chi-square tests and independent sample t-tests. Outcomes were proportions of given treatments, cumulative incidences of recurrence and overall mortality by care setting. Cumulative incidences of recurrence were calculated using the Cumulative Incidence Competing Risk (CICR) method considering death without recurrence as a competing event [22, 23]. Overall mortality was calculated using the Kaplan Meier method. Cox proportional hazard models were used to characterize the influence of care setting on recurrence risk and overall mortality. Covariates were included in the multivariate model if they were judged to be clinically relevant, and comprised age at diagnosis, comorbidity, histological grade, T stage and N stage. Treatments were not included in the multivariable model because one of the mechanisms through which oncogeriatric care influences outcomes is through treatment. All statistical tests were two-sided and a *p* value of < 0.05 was considered statistically significant.

## Results

Overall, 268 patients in the oncogeriatric cohort and 1932 patients in the standard cohort were included. Patient and tumour characteristics are shown in Table 1. Patients in the oncogeriatric cohort were younger (median age of 75.6 versus 78.1 years, *p* < 0.001), but had more comorbidity and polypharmacy was more frequent compared to patients in the standard cohort. Furthermore, patients in the oncogeriatric cohort more often presented with larger tumour (57.1% versus 49.8% T2 tumours, *p* = 0.026) and lymph node-positive disease (60.4% versus 31.6%, *p* < 0.001, Table 1).

**Table 1:** Patient and tumour characteristics by care setting

	Oncogeriatric care (*n* = 268)		Standard care (*n* = 1932)		*p* value*
	*n*	%	*n*	%	
Age, years (median, IQR)	75.7	(72.1–80.0)	78.1	(73.6–83.3)	** < 0.001**
Comorbidities (number)					< 0.001*
0–1	80	29.9	886	45.9	
2–4	134	50.0	842	43.6	
≥ 5	31	11.6	204	10.6	
Unknown	23	8.6	0	0	
Polypharmacy					< 0.001*
No	177	66.0	1620	83.9	
Yes	69	25.8	312	16.1	
Unknown	22	8.2	0	0	
T stage					**0.026**
1	115	42.9	969	50.2	
2	153	57.1	963	49.8	
N stage					** < 0.001**
Negative	106	39.6	1322	68.4	
Positive	162	60.4	610	31.6	
Histological type					**0.043**
Ductal	192	71.6	1405	72.7	
Lobular	40	14.9	200	10.4	
Other	36	13.4	327	16.9	
Histological grade (BR)					0.289*
I	44	16.4	272	14.1	
II	112	41.8	604	31.3	
III	87	32.5	397	20.5	
Unknown	25	9.3	659	34.1	
Hormone receptor expression					0.877*
ER+ and/or PR+	218	81.3	1323	68.5	
ER− and PR−	41	15.3	256	13.3	
Unknown	9	3.4	353	18.3	
Her2Neu overexpression					0.387*
No	150	56.0	857	44.4	
Yes	35	13.1	238	12.3	
Unknown	83	31.0	837	43.3	

All statistical tests were two-sided and a *p* value of < 0.05 was considered statistically significant

*IQR* interquartile range, *BR* Bloom Richardson, *ER* estrogen receptor, *PR* progesterone receptor

*p value excluding missing data

**Fig. 1:**
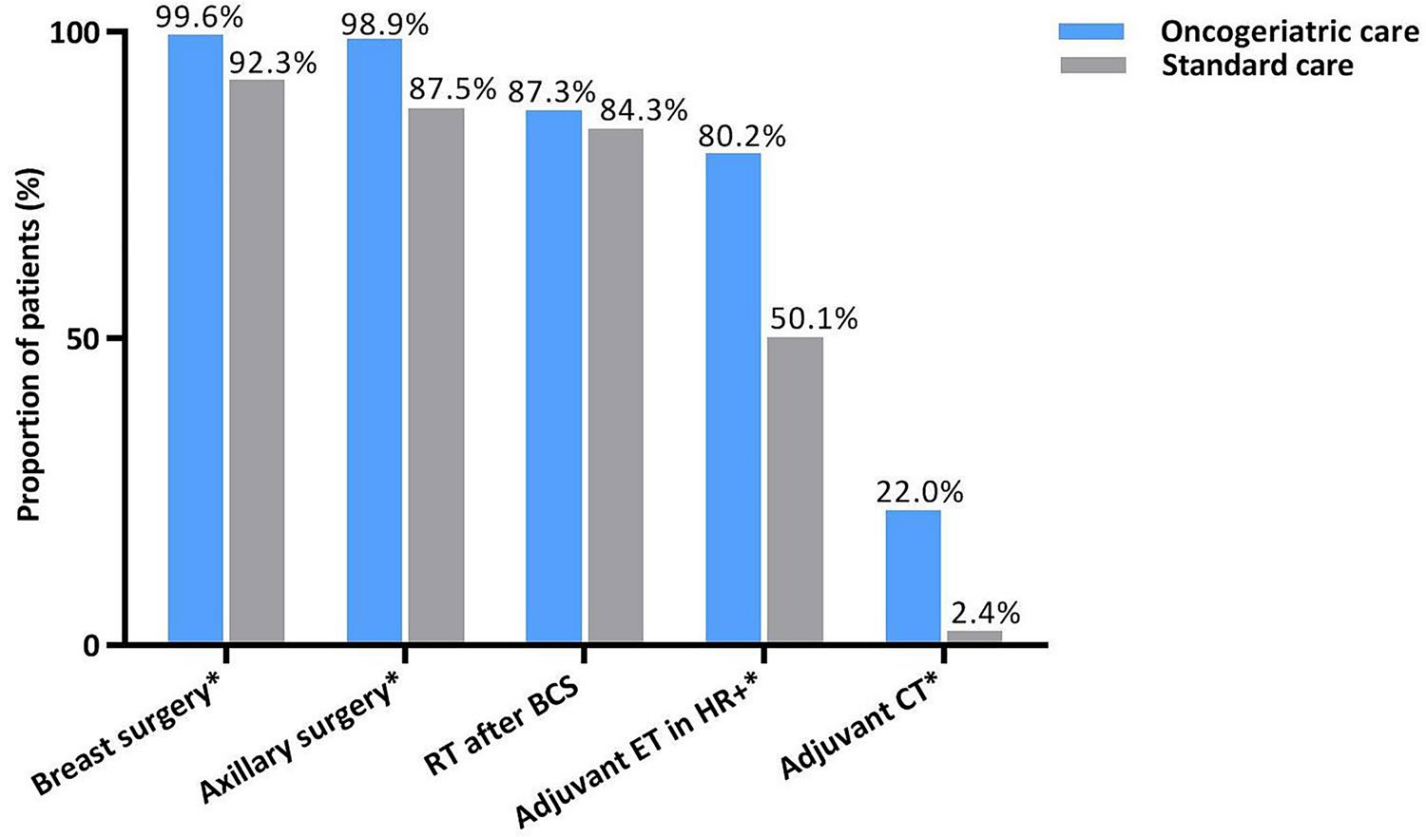
Treatments of given treatments by care setting. *Statistically significant difference. *RT* radiotherapy, *BCS* breast-conserving surgery, *ET* endocrine therapy, *HR* hormone receptor, *CT* chemotherapy

The majority of patients in the oncogeriatric cohort was seen in the SAOP (67.9%). Proportions of given treatments are shown in Fig. 1. Only one patient (0.4%) in the oncogeriatric cohort did not undergo surgery, compared to 149 patients (7.7%) in the standard cohort (*p* < 0.001). The predominant definitive surgery in the oncogeriatric cohort was BCS (55.8%) and mastectomy in the standard cohort (67.5%, *p* < 0.001). In surgically treated patients, axillary surgery was omitted less frequent in the oncogeriatric cohort than in the standard cohort (0.4% versus 12.5%, *p* < 0.001). Regarding adjuvant systemic treatments, a higher proportion of node-negative patients in the oncogeriatric cohort received adjuvant endocrine therapy (74.7% versus 32.2%, *p* < 0.001). Furthermore, a higher proportion of patients in the oncogeriatric cohort received adjuvant chemotherapy in both node-negative (16.0% versus 1.3%, *p* < 0.001) and node-positive patients (25.9% versus 4.8%, *p* < 0.001, Supplementary Table).

**Fig. 2:**
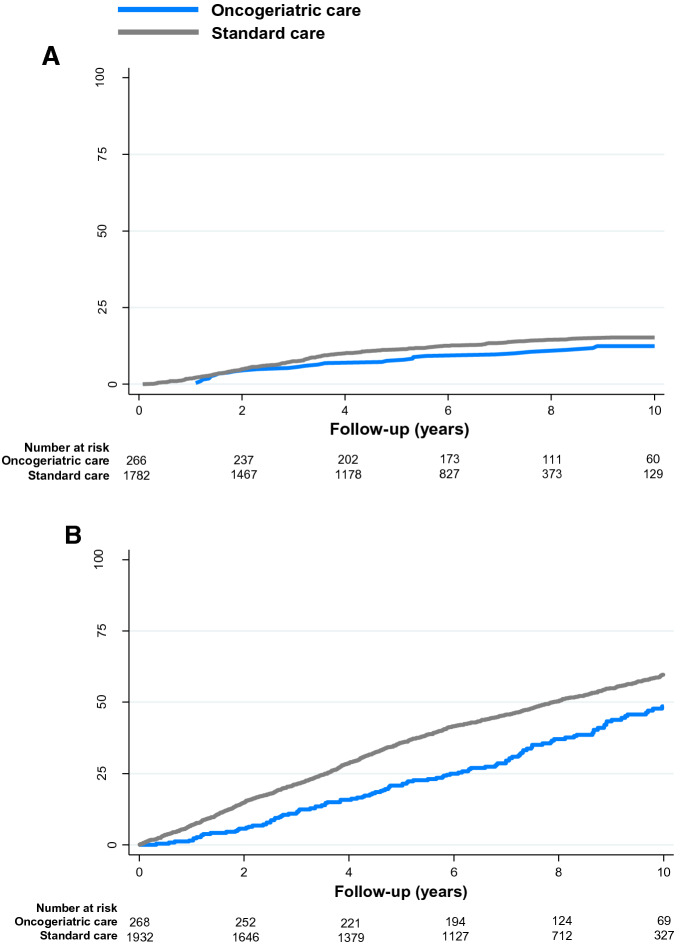
Cumulative incidence of recurrence and mortality by care setting. **A** Cumulative incidence of recurrence. **B** Overall mortality

Figure 2 graphically represents the cumulative incidences of recurrence and overall mortality by care setting. Median follow-up for recurrence was 7.2 years (IQR 4.3–9.7 years) in the oncogeriatric cohort and 5.6 years (IQR 2.7–7.7 years) in the standard cohort. Ten-year cumulative incidences of recurrence were 12.4% (95% CI 8.6–17.0%) in the oncogeriatric cohort and 15.2% (95% CI 13.5–17.1%) in the standard cohort. Oncogeriatric care was associated with a lower risk of recurrence (HR 0.67, 95% CI 0.46–0.98), which remained significant after adjustment for patient and tumour characteristics (adjusted HR 0.66, 95% CI 0.55–0.99, Table 2). Stratified by N stage, oncogeriatric care was associated with a lower risk of recurrence only in node-positive patients (HR 0.49, 95% CI 0.30–1.80), but this was no longer significant after adjustment for patient and tumour characteristics (HR 0.71, 95% CI 0.42–1.22, Table 2).

**Table 2:** Cumulative incidence of recurrence and mortality by care setting, and stratified by nodal stage

		10-year cumulative incidence % (95% CI)	HR (95% CI)	*p* value	Adjusted HR (95% CI)^a^	*p* value
Recurrence risk						
All patients	Standard care	15.2 (13.5–17.1)	1 (reference)	**0.037**	1 (reference)	**0.047**
	Oncogeriatric care	12.4 (8.6–17.0)	0.67 (0.46–0.98)		0.66 (0.44–0.99)	
Node-negative patients	Standard care	13.0 (11.0–15.2)	1 (reference)	0.373	1 (reference)	0.105
	Oncogeriatric care	11.0 (5.8–18.0)	0.76 (0.41–1.40)		0.56 (0.28–1.13)	
Node-positive patients	Standard care	19.9 (16.5–23.4)	1 (reference)	**0.004**	1 (reference)	0.216
	Oncogeriatric care	13.0 (8.1–19.1)	0.49 (0.30–0.80)		0.71 (0.42–1.22)	
Overall mortality						
All patients	Standard care	59.6 (57.1–62.1)	1 (reference)	** < 0.001**	1 (reference)	**0.001**
	Oncogeriatric care	48.3 (41.8–55.4)	0.66 (0.54–0.80)		0.69 (0.55–0.87)	
Node-negative patients	Standard care	56.3 (53.3–59.4)	1 (reference)	** < 0.001**	1 (reference)	**0.019**
	Oncogeriatric care	38.4 (28.7–50.1)	0.53 (0.37–0.74)		0.62 (0.42–0.93)	
Node-positive patients	Standard care	66.6 (62.3–70.7)	1 (reference)	** < 0.001**	1 (reference)	**0.027**
	Oncogeriatric care	54.7 (46.3–63.4)	0.64 (0.50–0.82)		0.72 (0.54–0.96)	

All statistical tests were two-sided and a *p* value of < 0.05 was considered statistically significant

*HR* hazard ratio

^a^Adjusted for age, comorbidity, histological grade and T stage. In addition, HRs in "All patients" were adjusted for nodal stage

For overall mortality, median follow-up was 7.7 years (IQR 5.2–10.0 years) for the oncogeriatric cohort and 6.8 years (IQR 3.5–9.2 years) for the standard cohort. Ten-year overall mortality was 48.3% (95% CI 41.8–55.4%) in the oncogeriatric cohort and 59.6% (95% CI 57.1–62.1%) in the standard cohort. Oncogeriatric care was associated with a lower overall mortality (HR 0.66, 95% CI 0.54–0.80), which remained significant after adjustment for patient and tumour characteristics (HR 0.69, 95% CI 0.55–0.87). Stratified by N stage, oncogeriatric care was associated with a lower overall mortality in both node-negative and node-positive patients, and both associations remained significant after adjustment for patient and tumour characteristics (adjusted HR of 0.62, 95% CI 0.42–0.93, in node-negative patients and adjusted HR of 0.72, 95% CI 0.54–0.96, in node-positive patients, Table 2).

## Discussion

In our study, patients treated in the oncogeriatric care setting received more adjuvant systemic treatments and had a lower risk of recurrence. Furthermore, overall mortality was lower for patients treated in the oncogeriatric care setting. However, the observed differences in patient and tumour characteristics between patients treated in the two care settings, preclude making firm inferences on the effect of care setting on the outcomes.

In contrast with a previous study that compared patients with stage IV breast cancer from the same cohorts, we observed several differences in patient and tumour characteristics, including younger age, more comorbidity, larger tumours and more node-positive disease in patient treated in the oncogeriatric care setting compared to the standard care setting [24]. Three main reasons could explain these differences. First, the higher comorbidity in the oncogeriatric cohort could (partly) be a result of more thorough examination for comorbidity as this is one of the aims of the SAOP. Second, the differences in patient and tumour characteristics could be explained by selection bias in the oncogeriatric cohort as this was a hospital-based cohort opposed to the standard cohort which was a regional population-based cohort. As a result, unlike patients in the standard cohort who were all patients who met the inclusion criteria in a certain geographic area, patients in the oncogeriatric cohort might be selectively (self) referred to the Moffitt Cancer Center. Possibly, patients with more advanced disease had a higher chance of being referred. Also, travel distance may have been a larger barrier in the oldest old as impaired mobility is more prevalent at this age. A third explanation could be that older patients with breast cancer in the US are different from their counterparts in the Netherlands due to differences in health care system and health care access between the two countries. As patients in the oncogeriatric cohort presented with more advanced disease, we considered differences in screening programme or participation rate. On the one hand, both countries had a screening programme of biannual mammography up to 75 years at the time, and if any difference in participation rate existed, the participation rate was in fact higher in the US because the upper age limit was raised from 69 to 75 years in the Netherlands in 1998, only 1 year before our study period. On the other hand, screening was likely more selective in the US due to out-of-pocket expenditures for preventive services despite Medicare insurance, whilst these expenditures were covered by mandatory insurance in the Netherlands [25]. Also outside of screening programmes, fee-for-service care in the US may prevent patients from seeking medical care causing later detection. This issue is less pronounced in the Netherlands because insurance is mandatory and has a wider coverage with less out-of-pocket expenditures.

### Treatments

Overall, patients treated in the oncogeriatric care setting received more extensive treatment. Omission of surgery was rare and more systemic treatment was administered compared to patients treated in the standard care setting. These findings are in contrast with the predominate finding in literature that a GA more often leads to a less extensive treatment [6]. However, the higher use of systemic treatments in the oncogeriatric cohort can be largely explained by the broader indications for endocrine therapy and chemotherapy in de US compared to the Netherlands [26–28]. Moreover, it was demonstrated that the Netherlands were more conservative in the administration of chemotherapy compared to other European countries, whilst more liberal in the omission of surgery compared to the US, probably beyond differences in guidelines [29–31]. As differences in guidelines and treatment culture between the US and the Netherlands likely contributed to the differences in treatments, it is unclear to what extent the oncogeriatric evaluation influenced treatment decisions.

### Outcomes

Patients treated in the oncogeriatric care setting had a lower risk of recurrence and lower overall mortality. Furthermore, stratified analyses suggest that the lower risk of recurrence was based on a lower risk amongst node-positive patients rather than node-negative patients. Unfortunately, as selection bias due to selective referral to the Moffitt Cancer Center and residual confounding due to unmeasured differences between patients in the US and the Netherlands are of concern, no firm conclusions can be made about the effect of care setting on outcomes. Of note, our findings do suggest that some older patients may benefit from more systemic treatments in terms of a lower risk of recurrence. Yet, it remains uncertain whether the oncogeriatric care contributed to these higher rates of systemic treatments, as we lacked data on treatment alterations that can be attributed to the geriatric evaluation. Finally, the follow-up interval difference in years two to five after diagnosis of 6 months in the oncogeriatric care cohort versus 12 months in the standard care cohort could potentially have led to a relative underestimation of recurrences in the latter. However, it is unlikely that this explains the difference in recurrence rates completely.

### Strengths and limitations

The strength of our study was that the study design allowed us to include relatively large numbers of patients compared to other studies on the effect of a GA on outcomes. Unfortunately, our study had important limitations. First, the observed differences in patients and tumour characteristics raised concern of selection bias due to selective (self) referral to the Moffitt Cancer Center and residual confounding due to unmeasured factors we could not adjust for. These issues precluded us from drawing firm conclusions about the effect of oncogeriatric care on outcomes. Second, information on the detection and management of non-oncologic conditions and treatment modifications in the oncogeriatric cohort were not available which could have alternatively provided insight in the impact of the oncogeriatric approach. A minor limitation was that some patients in the oncogeriatric cohort were not seen in the SAOP per se. However, as these patients were treated by oncologists who have done years of geriatric oncology training with the geriatric oncologists in the SAOP, we considered the whole cohort as oncogeriatric care. Lastly, information on quality of life and functional outcomes was not available, whilst these are important targets of oncogeriatric care.

### Conclusions

In conclusion, patients treated in the oncogeriatric care setting had a lower risk of recurrence than patients treated in the standard care setting, which may be explained by more systemic treatment. Overall mortality was also lower in patients treated in the oncogeriatric care setting, but other explanations besides care setting per se could not be ruled out as the cohorts had different patient profiles. Future studies need to clarify the impact of an oncogeriatric approach on outcomes, including not only survival but also quality of life and functional outcome measures.

## Electronic supplementary material

Below is the link to the electronic supplementary material.

Supplementary file1 (DOCX 17 kb)
