# Supplementary material for: Early stage breast cancer treatment and outcome of older patients treated in an oncogeriatric care and a standard care setting: an international comparison
Source: Breast Cancer Res Treat. 2020 Aug 19;184(2):519–26. doi: 10.1007/s10549-020-05860-7 (PMC7599178; doi:10.1007/s10549-020-05860-7)
Supplement: Supplementary file 1 — Supplementary file1 (DOCX 17 kb) [file 10549_2020_5860_MOESM1_ESM.docx]

| **Supplementary Table.** Treatment strategies by nodal status and care setting | | | | | | | | | | |
| --- | --- | --- | --- | --- | --- | --- | --- | --- | --- | --- |
|  | N0 (n=1428) | | | | | N1 (n=772) | | | | |
|  | Oncogeriatric care (n=106) | | Standard care (n=1322) | | p value | Oncogeriatric care (n=162) | | Standard care (n=610) | | p value |
|  | n | % | n | % |  | n | % | n | % |  |
| Breast surgery |  |  |  |  | **0.001** |  |  |  |  | **0.011** |
| Yes | 106 | 100 | 1204 | 91.1 |  | 161 | 99.4 | 579 | 94.9 |  |
| No | 0 | 0 | 118 | 8.9 |  | 1 | 0.6 | 31 | 5.1 |  |
| Most extensive breast surgery |  |  |  |  | **<0.001** |  |  |  |  | **<0.001*** |
| BCS | 62 | 58.5 | 458 | 38.0 |  | 87 | 54.0 | 121 | 20.1 |  |
| Mastectomy | 44 | 41.5 | 746 | 62.0 |  | 72 | 44.7 | 458 | 79.1 |  |
| Unknown | 0 | 0 | 0 | 0 |  | 2 | 1.2 | 0 | 0 |  |
| Axillary surgery |  |  |  |  | **<0.001** |  |  |  |  | **<0.001*** |
| Yes | 105 | 99.1 | 1043 | 86.6 |  | 159 | 98.8 | 517 | 89.3 |  |
| No | 1 | 0.9 | 161 | 13.4 |  | 0 | 0 | 62 | 10.7 |  |
| Unknown | 0 | 0 | 0 | 0 |  | 2 | 1.2 | 0 | 0 |  |
| Most extensive axillary surgery |  |  |  |  | **<0.001*** |  |  |  |  | **0.003*** |
| Sentinel lymph node biopsy | 81 | 77.1 | 452 | 43.3 |  | 25 | 15.7 | 41 | 7.9 |  |
| Axillary lymph nodes dissection | 24 | 22.9 | 591 | 56.7 |  | 133 | 83.7 | 476 | 92.1 |  |
| Unknown | 0 | 0 | 0 | 0 |  | 1 | 0.6 | 0 | 0 |  |
| Radiotherapy after BCS |  |  |  |  | 0.150 |  |  |  |  | 0.475 |
| Yes | 56 | 90.3 | 381 | 83.2 |  | 74 | 85.1 | 107 | 88.4 |  |
| No | 6 | 9.7 | 77 | 16.8 |  | 13 | 14.9 | 14 | 11.6 |  |
| Adjuvant endocrine therapy in HR+ |  |  |  |  | **<0.001** |  |  |  |  | 0.779 |
| Yes | 62 | 74.7 | 292 | 32.2 |  | 112 | 83.6 | 346 | 84.6 |  |
| No | 21 | 25.3 | 573 | 63.3 |  | 22 | 16.4 | 63 | 15.4 |  |
| Adjuvant chemotherapy |  |  |  |  | **<0.001** |  |  |  |  | **<0.001** |
| Yes | 17 | 16.0 | 17 | 1.4 |  | 42 | 26.1 | 29 | 5.0 |  |
| No | 89 | 84.0 | 1187 | 98.6 |  | 119 | 73.9 | 550 | 95.0 |  |
| SAOP assessment |  |  |  |  |  |  |  |  |  |  |
| Yes | 73 | 68.9 |  |  |  | 109 | 67.3 |  |  |  |
| No | 32 | 30.2 |  |  |  | 47 | 29.0 |  |  |  |
| Unknown | 1 | 0.9 |  |  |  | 6 | 3.7 |  |  |  |
| *p value excluding missing data. BCS: breast-conserving surgery, HR: hormone receptor. SAOP: Senior Adult Oncology Program. | | | | | | | | | | |
